# Ocular trauma from land mines among soldiers treated at a University Hospital in Medellín, Colombia

**Published:** 2013-12-31

**Authors:** Liliana Moreno, Luis F Velasquez, Carlos A Restrepo, Jose D Paulo, Jorge Donado, Marta L Muñoz, John J Aristizabal

**Affiliations:** 1 Departmento Ophthalmology. Pablo Tobón Uribe Hospital, Universidad Pontificia Bolivariana, Medellin, Colombia. lilymon9@hotmail.com; 2 Department of Surgery, Pablo Tobón Uribe Hospital. Universidad Pontificia Bolivariana, Medellin, Colombia. jdonado@hptu.org.co

**Keywords:** Eye injuries, trauma severity index, eye foreign bodies, penetrating ocular injuries

## Abstract

**Introduction:**

Currently ocular combat injuries are complex and associated with poor visual outcomes. Our objective is to characterize the military population that suffer land mine combat ocular trauma in Colombia and identify the type of wound, treatment and visual outcomes.

**Methods:**

Retrospectively review of medical history of soldiers evaluated in Pablo Tobon Uribe Hospital, whom had land mine trauma during January of 2004 and December 2012.

**Results:**

635 soldiers had land mine trauma, 153 of them had ocular trauma (226 eyes). Open ocular trauma was observed in 29.6%. The Ocular Trauma Score was calculated in 183 eyes, the initial visual acuity was not possible to be reported in the rest of them; the 45% of the eyes were classified in category 3. Three patients had no light perception in both eyes. 97.3% of the eyes received medical treatment and 49.1% had surgery also. Primary evisceration was made in 5.8% and enucleation in 1.8%. Intraocular foreign body was observed by ultrasonography in 11.1% and in 5.8% by orbital tomography. Eleven patients were legally blind at discharge.

**Conclusions:**

The ocular trauma related to a land mine is highly destructive at an ocular level. The treatments associated with better visual outcomes are primary closure of globe and systemic antibiotics; although the characteristics of the wound itself are the main prognostic factor. The Ocular trauma score is a useful tool for determining visual outcome in combat ocular trauma.

## Abstract (es)

**Introducción:**

Los combates armados generan heridas oculares complejas con mal pronóstico visual. Nuestro objetivo es caracterizar la población militar que sufre trauma ocular de combate en Colombia asociado a minas antipersona, así como las características de las lesiones, el tratamiento recibido y desenlace visual final.

**Métodos:**

Se evaluó retrospectivamente las historias clínicas de soldados atendidos en el Hospital Pablo Tobon Uribe, que sufrieron accidente por mina antipersona durante el período entre enero de 2004 y diciembre de 2012.

**Resultados:**

635 soldados, sufrieron trauma por mina antipersona; de estos, 153 (226 ojos) tuvieron trauma ocular. El 29.6% tuvieron trauma abierto. Se calculó el Ocular Trauma Score en 183 ojos. El 45% de los ojos se clasificaron como categoría 3. Tres pacientes tuvieron una visión final de no percepción de luz por ambos ojos. El 97.3% de los ojos tuvieron tratamiento farmacológico y 49.1% recibieron cirugía además. Se realizó evisceración primaria en el 5.8% y enucleación en 1.8%. Se logró comprobar cuerpo extraño intraocular por ecografía en el 11.1% y por tomografía de órbitas en el 5.8%. Once pacientes fueron legalmente ciegos, al momento de abandonar el hospital.

**Conclusiones:**

Las principales medidas terapéuticas asociadas con mejoría del pronóstico visual son el cierre primario de herida y la administración de antibióticos; aunque las características de las heridas oculares son el principal factor pronóstico. El Ocular Trauma Score es una herramienta útil para determinar el pronóstico visual en trauma ocular de combate.

## Introduction

The armed conflict in Colombia has lasted for over 50 years and has resulted in countless deaths and combat wounds. The evolution in warfare tactics has led to anti-personnel landmines and explosive devices (cluster munitions) becoming the leading causes of ocular combat trauma worldwide, other causes include wounds from firearms and vehicular accidents^1^. The incidence of ocular trauma from armed conflicts involving the United States ranged from 0.5 to 13.0%^2^. In our environment, little is known about the incidence and characteristics of ocular trauma that is being sustained by our soldiers. Likewise, little is known of the impact this has on rehabilitation efforts and on the disabilities that are generated.

Few large-scale military conflicts have been studied in the research literature worldwide, with the Vietnam War, the conflict in Iraq in 2003, and the Russian-Afghan war being the most studied^1^ ^-^ ^4^. These studies have been able to identify the factors that determine the visual prognosis and the salvaging of the organ especially in cases of penetrating trauma, which are the most serious and most common in the warfare environment. Of these, 91% are patients that are left with a vision of less than 20/800^1^. The Ocular Trauma Score (OTS)^4^ was first published in 2002 and it estimates visual function six months after the trauma by assigning a score according to the initial visual acuity and then subtracting another score according to the presence of eyeball rupture, endophthalmitis, ocular perforation, retinal detachment and afferent pupillary defect. Patients are classified into five categories, with category 1 and 2 having the worst visual prognosis. This system has been validated in studies of patients with ocular trauma from war^5^. It was been further determined that primary closure and systemic antibiotics are the measures associated with improved visual prognosis. The timing of the removal of intraocular foreign bodies is of little importance and it is generally unrelated to a worsened prognosis^3^ ^,^ ^6^.

This study is directed toward characterizing the military population that suffers combat-related ocular trauma associated with landmine explosions in Colombia, and, similarly, characterizing the wounds, the processing received and the final visual outcome over a period of eight years at a level four hospital and trauma referral center.

## Materials and Methods

An observational, descriptive study was conducted that included a review of medical records of patients treated for trauma from landmines at the Pablo Tobon Uribe Hospital in Medellin (Referral Center for the armed forces of the region and the country, with 370 beds and level-four care) from January 2004 to December 2012.

Data extracted from the medical records included: age, sex, affected eye (left and/or right), type of injury, diagnostic aids used (orbital computed axial tomography (CAT), ocular ultrasound) treatment conducted (medical and surgical), length of hospital stay (1-7 days 8-14 days 15-30 days more than 30 days), presence or absence of an afferent pupillary defect in the initial evaluation and at the time of discharge (alive or dead).

Ocular lesions were classified according to the BETTS (Birmingham Eye Trauma Terminology System)^7^ in closed trauma that included contusions (no full-thickness wound to the eye wall) and lamellar lacerations (injury of a partial thickness of the eye wall); and for open trauma, they are comprised of penetrating injuries (a lacerating wound that penetrates the eyeball), piercing (wound with entrance and exit orifices caused by the same object), intraocular foreign body (IOFB, classifies a part of the penetrating wound for its effect on visual prognosis) or ocular rupture (usually caused by blunt force trauma that elevates intraocular pressure until explosion or rupture of the eyeball with a great loss of tissue). The compromising of eyelids and attachments were classified as palpebral edema, laceration, injury or lack of compromise to the eyelids.

The Ocular Trauma Score (OTS) is a method developed to determine the visual outcome after 6 months among patients with ocular trauma. It uses as categorical parameters the initial visual acuity and the type of injury sustained and it provides a single score (Table 1). Patients were grouped into five groups according to their score and percentages were calculated for the final visual acuity as shown in Table 2. Higher scores indicate a better visual prognosis. It is useful for predicting the visual range in which the patient will be in 6 months after the trauma, and it also serves as a tool to identify serious eye injuries and to counsel both families and the patient^2^ ^,^ ^4^ ^,^ ^8^.

**Table 1:**
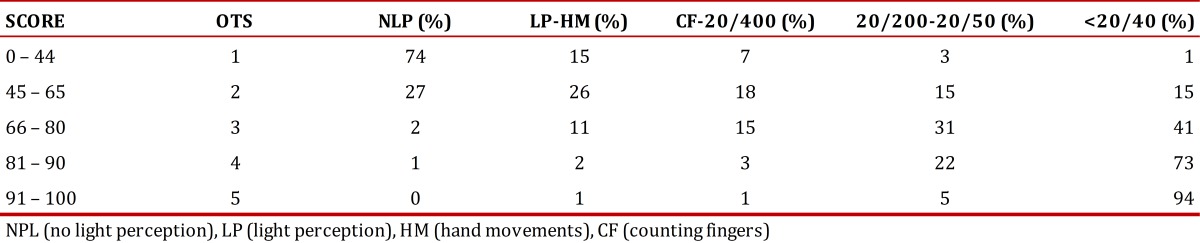
Calculating the Ocular Trauma Score

**Table 2:**
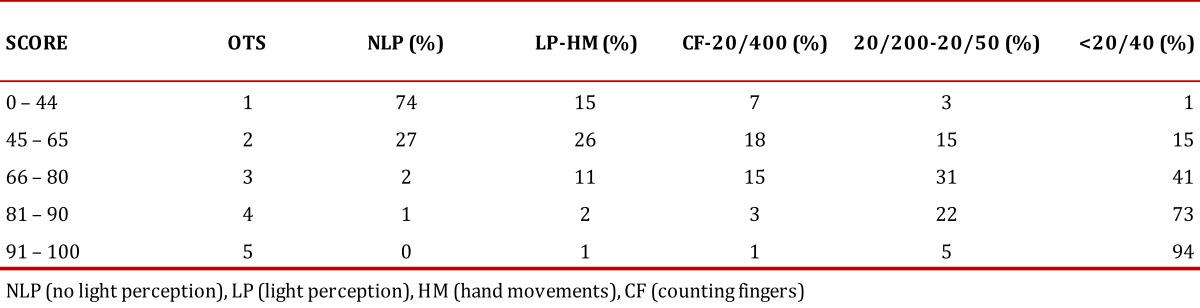
Calculation of final visual acuity by Ocular Trauma Score group

The diagnostic aids utilized along with the results were analyzed. In the orbital computed axial tomography the presence of orbital fractures were reported as: no fracture, fracture of the orbital floor, fracture of the orbital roof, fracture of the medial orbital wall, fracture of the lateral orbital wall, fracture of the medial wall and floor of the orbit, with fracture of the medial wall and orbital roof, with three broken orbital walls, with all orbital walls broken or IOFB. Meanwhile, for ocular ultrasound it was recorded whether or not there was a cataract, as well as whether or not there was vitreous opacity, retinal detachment, IOFB or others.

The type of treatment received was determined and classified as: drug treatment, debridement of the lamp slit, exploration in the operating room without finding injuries, exploration in the operating room with primary repair of wounds, evisceration, enucleation or eyelid injury repair.

For statistical analysis, categorical variables are presented as absolute and relative frequencies and continuous variables are presented as the arithmetic mean and standard deviation. The SPSS version 15 statistical package was used. 

The research was conducted according to the principles of the Declaration of Helsinki, 2008 (Seoul, Korea), resolution 008430/1993 of the Colombian Ministry of Health and the study was approved by the Research Ethics Committees at both the Pablo Tobon Uribe Hospital and at the School of Health Sciences at the Universidad PontificiaBolivariana.

## Results

Of the 635 patients affected by anti-personnel mines in our institution during the 8 year period of 2004-2012, a total of 226 eyes from 153 patients (23.9%) suffered some type of eye injury and were included in the study. The average age was 24.8 ± 4.3 years (SD), all patients were men, and all were soldiers from the Colombian Army. Forty-one and four-tenths percent or 63 patients were hospitalized for a period between 1-7 days, and only 13.1% (20) were there for more than 30 days.

The number and percentage of eyes studied according to the type of injury is shown in Table 3. The total of eyes with an open eye wound was 67 (29.6%) and bilateral in 74 (48.7%) of 152 patients.

**Table 3:**
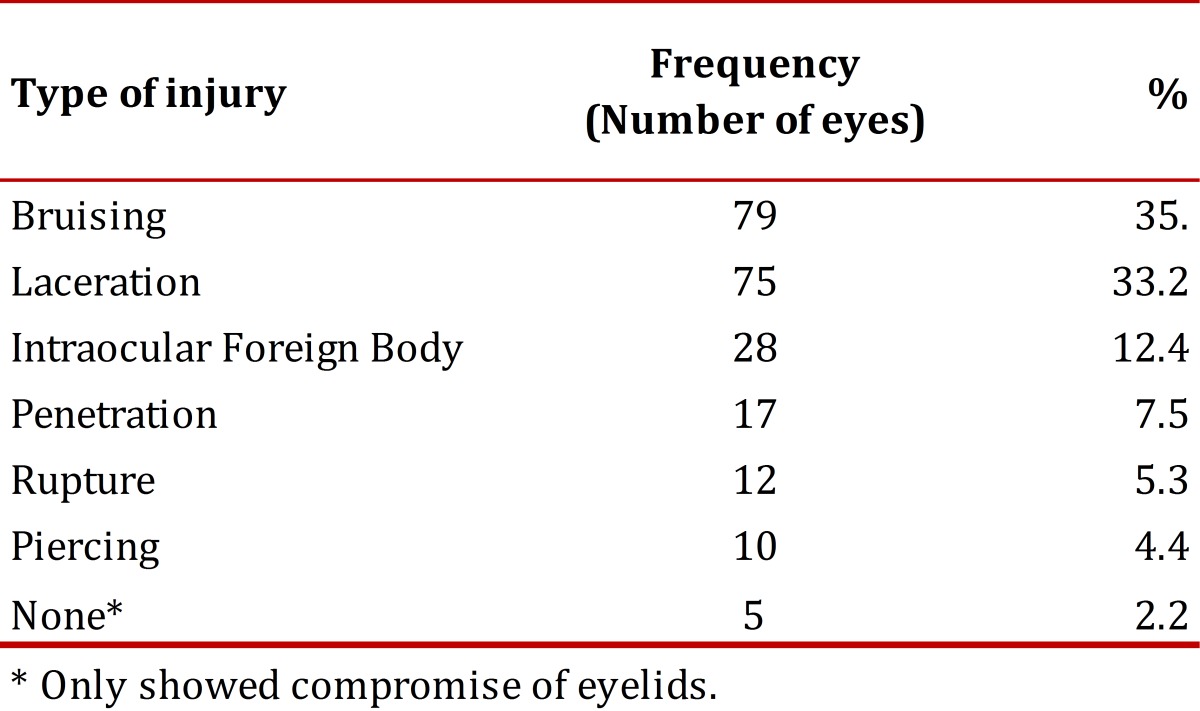
Eye injuries

Visual acuity was classified into groups for greater convenience. The majority of eyes had an initial visual acuity (the first occasion that could be taken often several hours after the trauma occurred due to delay in transport from the battlefield or due to the state of patient consciousness) of 20/400 or the movement of hands. At hospital discharge, the visual acuity most frequently found was 20/40 or as better measured by Snellen´s Chart, which does not necessarily reflect the final vision of the patients since 21 (9.3%) of 152 required subsequent intervention for which they were sent to another institution to finish their treatment.

 It was not possible to monitor visual acuity after discharge in most patients Table 4.

**Table 4:**
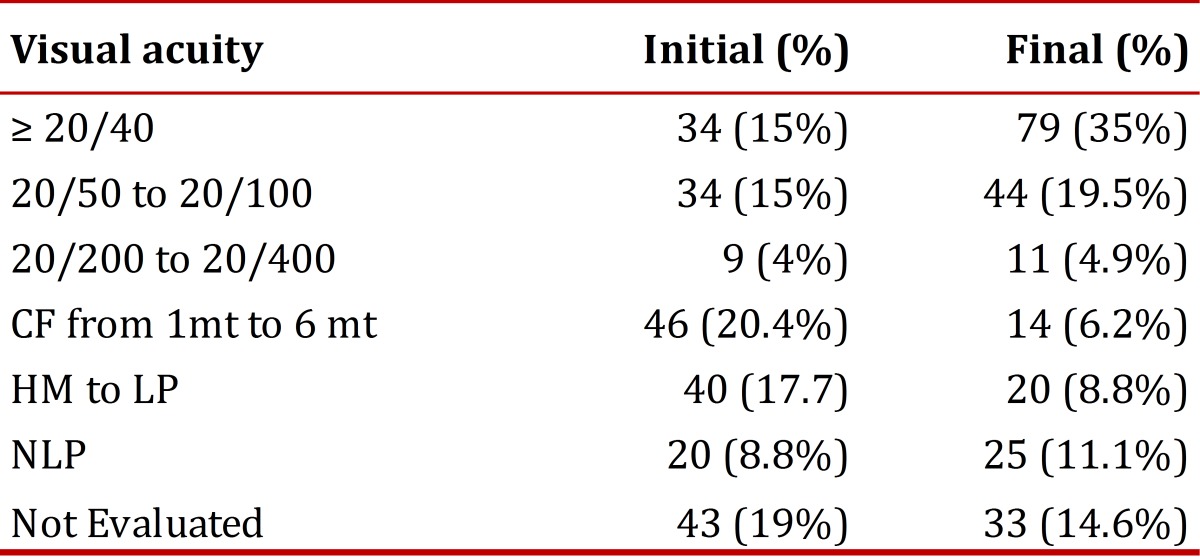
Initial and final visual acuity

The number of patients that were legally blind (vision equal to or worse than 20/200 in the best eye) at the initial evaluation was 31 (13.7%) and at discharge it was 11 (4.8%); three were left with an acuity of not perceiving light from either eye. In some cases, it was not possible to document the final visual acuity since 33 eyes of 25 patients required surgical management at other institutions and were thus lost to follow-up, or died. For an altered state of consciousness present on admission for 28 patients (43 eyes), it was not possible to determine initial visual acuity or to carry it out in a standardized way; in 8 (3.5%) of 152 patients the data was not obtained on admission or on discharge.

In 183 eyes, an OTS was possible to determine with the finding that most were located in group 3 (85 eyes) and 7 eyes were in group 1 Table 5. Only one patient had endophthalmitis requiring intravitreal antibiotics during hospitalization, 8 patients had retinal detachment and, due to the nature of trauma, in 44 eyes it was not possible to determine the presence of DPA needed for calculating the OTS. The final visual acuity percentage was calculated according to the OTS group taking into account that this final vision score refers to the last reported during hospitalization, which in most patients was for less than 7 days.

**Table 5:**
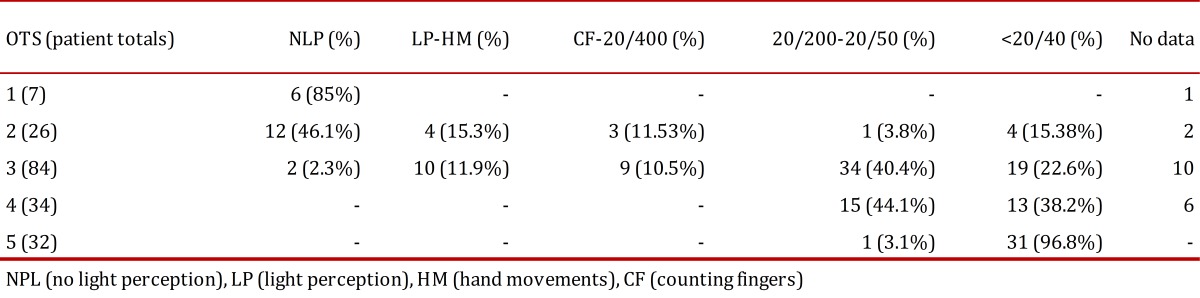
Classification by Ocular Trauma Score and final Visual acuity (VA)

For patients classified by OTS, the probability for having a favorable final vision was calculated, which was understood as a visual acuity equal to or better than that used in counting fingers. An unfavorable final vision was viewed as vision worse than that used in counting fingers (Table 6). It was found that patients with open eye wounds had a greater probability of having an unfavorable visual outcome for all OTS groups. For OTS 1, 100% had an unfavorable visual outcome; for OTS 2, the Odds Ratio (OR) was 16 (1.07-512.7) *p*= 0.02; for OTS 3, the OR was 8.33 (1.84-40.5) *p*= 0.00 are unfavorable; and for OTS 4 and 5 it was 0%.

**Table 6:**
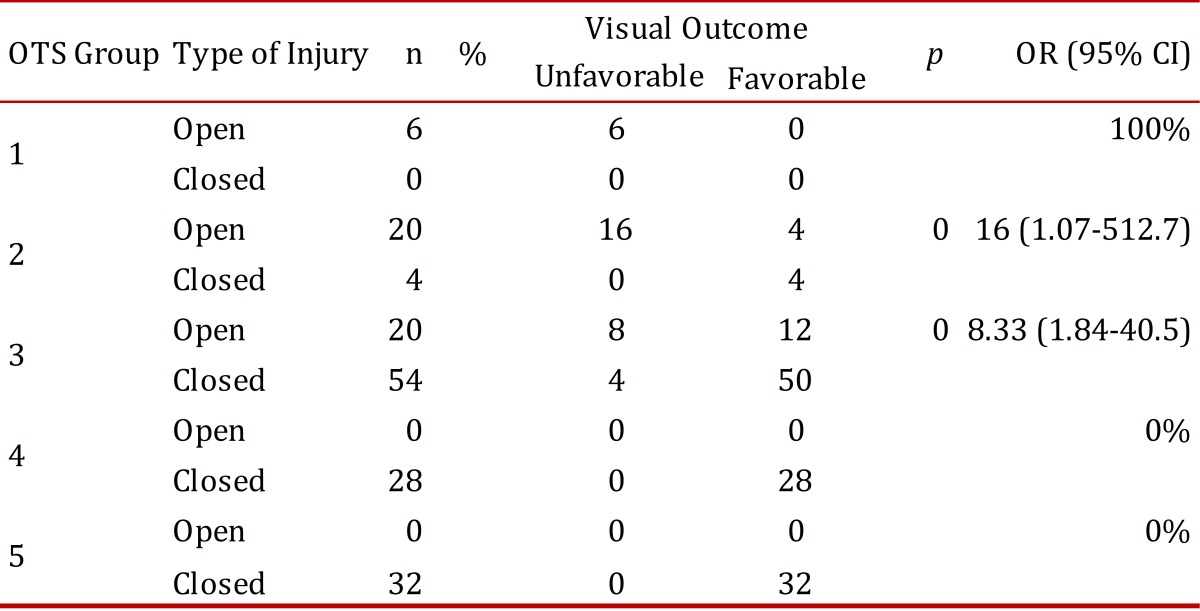
Unfavorable visual outcome by type of eye injury as classified by the Ocular Trauma Score

There was surgical treatment for 111 (49.1%) eyes from 59 patients. A total of 220 (97.3%) eyes required pharmacological management. Only 43 (19.0%) eyes required debridement in a slit lamp. Of the eyes examined, 12 (5.3%) had no reparable injury while 41 (18.1%) eyes presented wounds that were sutured. Due to the impossibility of reconstruction, 13 (5.8%) eyes were eviscerated and 4 (1.8%) were enucleated. In 153 (67.7%) eyes it was further reported that an eyelid injury occurred; of these, 41 (18.1%) required surgical correction.

The afferent pupillary defect in the first evaluation was present in 16 (7.1%) of the affected eyes, and was absent in 104 (46.0%). It was not possible to be evaluated in 48 (21.2%) cases; the data consigned to the medical record was not found for 58 (25.7%) of the 226 eyes evaluated.

Orbital computed axial tomography (CAT) was performed on 85 eyes (37.6%). There was no significant finding as to fracture or IOFB in 24 (10.6%) of the eyes examined; 11 (4.9%) evidenced an orbital wall fracture and 6 (2.7%) had a fracture on 2 or more orbital walls. IOFB was detected in 13 (5.8%) eyes by means of the CAT. Ocular ultrasonography was performed on 102 eyes (45.1%); of these, 7 (3.1%) gave normal results, 13 (5.8%) showed a cataract, 33 (14.6%) showed vitreous opacity, 10 (4.4%) revealed retinal detachment, and 25 (11.1%) showed IOFB.

## Discussion

The armed conflict in Colombia has left countless dead and wounded and untold damage, not only to the soldiers and civilians affected, but also to their families. The Assembly of the World Health Organization recognized the issue of landmines as a public health problem in 1998. They cause multiple injuries through the complex mechanisms of trauma, highly contaminated wounds, prolonged hospital stays, disability and sequelae. Colombia has the highest number of victims in the world from land mines; it is the country with the greatest number of mines implanted in the soil that affects 46% of the country's municipalities and 31 of the 32 departments^9^. Antioquia has the highest number of cases in the country with 19.8% of the total. 40% of accidents and incidents involving landmines are related to military clearance activity^10^. 

Facial exposure makes the eyes an easy target for injuries from explosive devices. Ocular trauma can be derived from many forms of military assault but the most common and severe are related to detonating objects (mines, grenades, etc.)^11^ ^-^ ^16^. From our series, 23% of landmine victims showed ocular involvement. This is a slightly higher number than was reported in U.S. armed conflicts^2^, but is similar to that reported by the International Committee of the Red Cross in 1991^17^, or to those reported by Restrepo*et al*., in a study conducted at our institution between the years 2003-2005 where the types of injuries found in patients who were victims of landmines were evaluated. It was found that 94.7% of victims were soldiers and that ocular trauma was reported for 25.8%^9^ of the patients. Ocular trauma from combat remains a challenge for the trauma ophthalmologist as different situations influence the visual prognosis of patients. This is particularly the case in the combat environment and more so as it relates to landmines where more complex injuries are seen. They are characterized by having a high percentage of both metallic and organic intraocular foreign bodies, greater contamination from occurring mostly in rural areas, delay in receiving medical care due to the time lag in transfers from remote sites of difficult access, the possibility of sympathetic ophthalmia, among many others. Advances in technology and with the microsurgical instruments on which we currently rely have increased the chances of adequate reparation of eye injuries, as well as the possibility of saving the organ. However, it has been observed that the prognostic predictors for salvaging organs after ocular traumas from combat depends more on the characteristics of the wounds themselves than on their management^3^ ^,^ ^4^.

The main objectives in managing eye trauma from combat are primary closure of the wound and the administration of antibiotics. Studies of patients in Iraq found that delays in the removal of intraocular foreign bodies, independent of their nature (metallic, vegetable, plastic, etc.) was not a predictive factor for a poor visual prognosis^18^ ^,^ ^19^. In a related study, it was also found that the time at which the surgery was performed was also not related to the final visual acuity and it was found that the risk of endophthalmitis after open trauma with a metallic IOFB was low at 4%^20^.

Prognostic factors have been described for organ loss from metallic IOFB as wounds being >10 mm, retinal compromise, afferent pupillary defect, low initial visual acuity (<20/200), trauma from blunt objects or shrapnel, organic IOFB^1^ ^,^ ^7^ ^,^ ^20^.

Some of the above factors are included within the parameters for classification of the eye injuries by OTS. We found that the percentages of visual acuity at the end of hospitalization, discriminated by the OTS group calculated for 183 eyes Table 5, was correlated with the initial percentages reported for OTS for 6 months^4^; we assume that in most cases of combat-related ocular trauma the final visual potential could be defined at the end of hospitalization. After receiving initial treatment, suggested as primary wound closure and the administration of systemic antibiotics, if other conditions do not occur, such as endophthalmitis or retinal detachment after discharge, the final visual acuity was not expected to vary significantly. Other studies of combat-related ocular trauma have validated the use of the OTS as a tool for determining a visual prognosis^2^ ^,^ ^8^ ^,^ ^21^.

Orbital tomography and ocular sonography provide useful information for open ocular trauma, such as the presence of IOFB, orbital fractures or retinal detachments. In our study, orbital tomography was performed on 85 eyes (37.6%) and ultrasound was utilized on 102 eyes (45.1%). 

When tomography was used, IOFB was identified in 15.2% of cases, while ultrasound was used for detecting them in 24.5% of the eyes. In 6 patients both tests were used and IOFB was successfully identified by both methods in all cases. In several reports most cases of IOFB from combat trauma have been found to come from metal^19^ ^-^ ^22^, and while scanning is a diagnostic exam with great capacity to detect IOFB, it is limited to metal objects. This may explain the differences found with ultrasound in our study.

The overall rate of evisceration in our study was 5.75% and was 1.7% for enucleation; 28.5% of patients classified as category 1 on the OTS wereeviserated and 14.2% were primarily enucleated. In other studies of ocular trauma from combat, similar rates of organ loss were found^2^ ^,^ ^5^ ^,^ ^20^ ^,^ ^23^. While the secondary rate of evisceration/enucleation is not reported due to the lack of follow-up and monitoring, it could be assumed that as a measure to reduce the risk of sympathetic ophthalmia, all patients in category 1 of the OTS (final VA NLP) would end in evisceration/enucleation before 14 days^1^; however, Savar*et al.,* report that the close monitoring of these patients could be conducted to determine the appropriate time to perform a secondary enucleation with less psychological impact on the patient^23^. The remainder of the eviscerated/enucleated eyes was from OTS category 2 or could not be classified. Three patients had bilateral loss of the eyeball and 2 died. This indicates that the more severe the ocular trauma, the greater the probability of severe systemic lesions that may compromise the patient's life^1^ ^,^ ^21^.

The prevalence of endophthalmitis was reported as 0.4%, which is lower than that observed in other studies of combat trauma^7^ ^,^ ^20^. In the study conducted at the Pablo Tobon Uribe Hospital where tissue samples from patients affected by landmines were grown, it was reported that the most frequently found bacteria were *Pseudomonas aeruginosa* (13.2%) and *E. coli* (9.6%)^10^. Although the wounds from land mines are highly contaminated, we did not find high rates of endophthalmitis, which is probably due to coverage with systemic and topical antibiotics that includes the gram negative spectrum.

## Conclusions

Combat eye injuries are complex from their high frequency of IOFB and from contamination for which the visual prognosis is mainly related to the characteristics of those injuries and to a lesser extent to the processing received. However, this does not ignore the importance of primary wound closure and administration of systemic antibiotics with the gram negative spectrum as therapeutic measures for improving the prognosis. In the referral sites for ocular trauma, the initial classification of injuries with the BETTS should be implemented to make appropriate use of diagnostic tools in suspected cases of IOFB, DR or orbital fractures. Despite the sensitivity of orbital ACT and ocular ultrasound, and while they are not sufficiently high to be used as a screening method, their clinical usefulness is significant. A better visual prognosis has generally been observed in cases of closed traumas versus those of open trauma.

The visual prognosis determined by the OTS classification is useful in cases of severe trauma for counseling patients and families since in group 1 and 2 cases there is a high probability of both primary and secondary organ loss.
